# Decreased expression of BRCA1 in SK-BR-3 cells is the result of aberrant activation of the GABP Beta promoter by an NRF-1-containing complex

**DOI:** 10.1186/1476-4598-10-62

**Published:** 2011-05-24

**Authors:** Crista Thompson, Gwen MacDonald, Christopher R Mueller

**Affiliations:** 1 Pathology and Molecular Medicine Department, Queen's University, Kingston, Ontario, Canada; 2 Biochemistry Department, Queen's University, Kingston, Ontario, Canada; 3 Queen's Cancer Research Institute, Queen's University, Kingston, Ontario, Canada; 4 Current Address: Friedrich Miescher Institute for Biomedical Research, Maulbeerstrasse 66, CH-4058 Basel, Switzerland

## Abstract

**Background:**

BRCA1 has recently been identified as a potential regulator of mammary stem/progenitor cell differentiation, and this function may explain the high prevalence of breast cancer in *BRCA1 *mutation carriers, as well as the downregulation of BRCA1 in a large proportion of sporadic breast cancers. That is, loss of BRCA1 function results in blocked differentiation with expansion of the mammary stem/progenitor cells. Because BRCA1 also maintains genomic integrity, its loss could produce a pool of genetically unstable stem/progenitor cells that are prime targets for further transforming events. Thus, elucidating the regulatory mechanisms of *BRCA1 *expression is important to our understanding of normal and malignant breast differentiation.

**Results:**

Loss of BRCA1 expression in the ErbB2-amplified SK-BR-3 cell line was found to be the result of loss of activity of the *ets *transcription factor GABP, a previously characterized regulator of BRCA1 transcription. The expression of the non-DNA binding GABPβ subunit was shown to be deficient, while the DNA binding subunit, GABPα was rendered unstable by the absence of GABPβ. Deletion analysis of the GABPβ proximal promoter identified a potential NRF-1 binding site as being critical for expression. Supershift analysis, the binding of recombinant protein and chromatin immunoprecipitation confirmed the role of NRF-1 in regulating the expression of GABPβ. The siRNA knockdown of NRF-1 resulted in decreased GABPβ and BRCA1 expression in MCF-7 cells indicating that they form a transcriptional network. NRF-1 levels and activity did not differ between SK-BR-3 and MCF-7 cells, however the NRF-1 containing complex on the GABPβ promoter differed between the two lines and appears to be the result of altered coactivator binding.

**Conclusions:**

Both NRF-1 and GABP have been linked to the regulation of nuclear-encoded mitochondrial proteins, and the results of this study suggest their expression is coordinated by NRF-1's activation of the GABPβ promoter. Their linkage to BRCA1, a potential breast stem cell regulator, implies a connection between the induction of mitochondrial metabolism and breast differentiation.

## Background

BRCA1 has been implicated in functions such as DNA repair, cell-cycle checkpoint control, protein ubiquitinylation, chromatin remodelling and transcriptional regulation (for reviews see [1,2]). However, the discovery that BRCA1 is required for mammary stem/progenitor cell differentiation [3] has cast BRCA1 in a different light. Mammary stem cells produce two cell populations - the inner luminal epithelial cells which express low molecular weight cytokeratins and estrogen receptor α (ERα), and the outer supporting basal myoepithelial cells which express high molecular weight cytokeratins and smooth muscle markers [4]. Liu *et al*. (2008) demonstrated that knockdown of BRCA1 in both *in vitro *and mouse model systems causes an increase in the stem/progenitor and myoepithelial cell populations (ERα-negative), and a decrease in the differentiated luminal epithelial cell population (ERα-positive). These results are consistent with the fact that BRCA1 activates ERα gene expression [5], and indicate that BRCA1 expression is required for the differentiation of mammary stem/progenitor cells into luminal epithelial cells and its loss results in blocked differentiation with expansion of the stem/progenitor cells [3]. Because BRCA1 also functions in maintaining genomic integrity (reviewed in [6]), these cells are more likely to progress to malignancy. Characterization of epithelial subpopulations in preneoplastic tissue from *BRCA1 *mutation carriers identified an aberrantly expanded luminal progenitor cell population as the likely target of transformation [7]. This model is also consistent with clinical data, *i.e*. the vast majority of breast tumours in women with germ-line mutations in *BRCA1 *display a basal-like (stem cell-like) phenotype characterized by a lack of expression of ER, PR and ErbB2 and robust expression of markers of myoepithelial differentiation [8]. Thus, there is strong evidence to suggest that loss of BRCA1 generates a cancer stem cell capable of initiating and driving breast tumour formation.

While mutational inactivation of *BRCA1 *in some familial breast and ovarian cancer is seen [9], a consistent pattern of *BRCA1 *gene mutation has not been identified in sporadic breast tumours [10-12]. However, decreased *BRCA1 *expression is observed in sporadic breast tumours, with decreasing expression correlating with increasing tumour grade [13-15]. This suggests that BRCA1 downregulation in sporadic cancer may also lead to a block in stem cell differentiation with the attendant increase in cancer risk.

The transcriptional regulation of the BRCA1 gene is complex with a variety of transcription factor binding sites having been identified (reviewed in [16]). Our previous analysis of the BRCA1 promoter had pointed to the *ets *transcription factor GA Binding Protein (GABP) and its RIBS binding element as key regulators of BRCA1 expression, particularly as it relates to its decrease in sporadic breast cancers [17]. The SK-BR-3 cell line, which overexpresses ErbB2, is known to have particularly low levels of BRCA1 protein. In this study, the BRCA1 promoter was shown to be less active in SK-BR-3 cells and the activity of the GABP protein was shown to be compromised. GABP is comprised of two distinct and unrelated subunits - GABPα, which contains the DNA-binding domain, and GABPβ, which contains the nuclear localization signal and transcriptional activation domain [18-21]. The expression of the GABPβ gene was shown to be decreased in SK-BR-3 cells and is in turn regulated by Nuclear Respiratory Factor-1 (NRF-1) [22]. While NRF-1 levels and activity are similar between MCF-7 and SK-BR-3 cells, the NRF-1 specific complex was altered suggesting that a coactivator interacting with NRF-1 differs between the two lines. BRCA1 expression appears to be regulated by a transcriptional network consisting of NRF-1 and GABP.

## Methods

### Cell culture

The human breast carcinoma cell lines MCF-7, T-47D, SK-BR-3, ZR75-1 and MCF-10A were obtained from the ATCC (Manassas, VA, USA), while 184hTERT cells [23] were a generous gift of Dr. Calvin Roskelley. MCF-7 and T-47D cells were maintained as previously reported [24]. ZR75-1 cells were maintained as per MCF-7/T-47Ds. SK-BR-3 cells were maintained in Dulbecco's modified Eagle's medium (Sigma, Oakville, Canada) supplemented with 10% fetal bovine serum (HyClone, Logan, UT, USA), 100 μg/mL streptomycin (Sigma) and 100 units/mL penicillin (Sigma). MCF-10A cells were maintained in DMEM F12 with L-Glutamine (HyClone) supplemented with 5% horse serum (Invitrogen, Burlington, Canada), 20 ng/mL epidermal growth factor (Invitrogen), 10 μg/mL insulin (Sigma), 0.5 μg/mL hydrocortisone (Sigma), 100 ng/mL cholera toxin (Sigma), 100 units/mL penicillin and 100 μg/mL streptomycin. 184hTERT cells were maintained in Clonetics® MEBM medium supplemented with Clonetics® SingleQuot, 400 μg/mL G418 (BioShop, Burlington, Canada), 1 μg/mL transferrin (BD, Mississauga, Canada) and 1.25 μg/mL isoproterenol (Sigma). Cells were cultured in a humidified atmosphere at 37°C and 5% CO_2 _.

### DNA constructs

Creation of L6-pRL has been previously described [24]. To create the FLAG-GABPα construct, the human GABPα gene was PCR amplified using pCAGGS-E4TF1-60 (obtained from Hiroshi Handa, [25]) as the template with the primers specified in Additional File 1. The GABPα PCR product was cloned into the pSCT-Gal vector using the restriction enzymes XbaI/HindIII. The pSCT-Gal-GABPα construct was then digested with HindIII, filled-in by Klenow, and then cut with XbaI. The p3×FLAG-CMV-10 vector (Sigma) was cut with BamHI, filled-in using Klenow, and digested with XbaI. The complete FLAG-GABPα construct was obtained by ligation of these two fragments. To generate the FLAG-GABPβ construct, the human GABPβ gene was PCR amplified using pCAGGs-E4TF1-53 (obtained from Hiroshi Handa, [25]) as the template with the primers specified in Additional File 1. The GABPβ PCR product was then cloned into the pMAL-c2 vector (New England Biolabs (NEB), Pickering, Canada) using the restriction enzymes XbaI/SalI. Isopropyl β-D-1-thiogalactopyranoside (IPTG)/Xgal colour screening was used to select positive clones. The GABPβ fragment was then cut out of the pMAL-c2 vector using SalI, filled in by Klenow, and subsequently digested using XbaI. The p3×FLAG-CMV-10 vector was prepared by first cutting with BamHI, followed by a Klenow fill-in reaction, and then digestion using XbaI. The final FLAG-GABPβ construct was generated by the ligation of these two fragments. pTRE-tight-GABPβ was prepared by digesting FLAG-GABPβ with SacI (partial) and XmaI and cloning the FLAG-tagged GABPβ sequence into pTRE-tight (Clontech, Mountain View, CA, USA).

To create the NRF-1 expression vector, pTRE-tight-GABPβ was digested with BglII and MluI to remove the GABPβ sequence, but retain the FLAG sequence. The human NRF-1 coding sequence was PCR amplified from pSG5-NRF-1 [26], a generous gift of RC Scarpulla, using the primers specified in Additional File 1. The PCR product was digested with BglII and MluI and cloned into pTRE-tight with the FLAG sequence to create pTRE-tight-NRF-1. The FLAG-tagged NRF-1 sequence was cut from pTRE-tight-NRF-1 using SacI and XbaI and cloned into p3×FLAG-CMV-10 vector to create the NRF-1 expression vector, p3×FLAG-NRF-1.

The *GABPβ *proximal promoter sequences were PCR amplified using the primers and templates specified in Additional File 1. The promoter regions were cloned into the pRL-null reporter plasmid (Promega, Madison, WI, USA) using the restriction sites indicated in Additional File 1. Gb-270 multimer was prepared by cloning double-stranded oligonucleotides comprised of 3 repeats of Gb-270 (sequence specified in Figure 6) with HindIII (5') and KpnI (3') overhangs into pRL-null containing a TATA box derived from the albumin gene and a G-free cassette.

### Recombinant NRF-1

The human NRF-1 coding sequence was PCR amplified from pSG5-NRF-1 [26] using the primers specified in Additional File 1. The PCR product was digested and cloned into the BamHI and HindIII sites of pMAL-c2. The recombinant protein was expressed and purified according to the manufacturer's protocol. The purified protein was eluted with 10 mM maltose in nuclear dialysis buffer (10 mM HEPES pH 7.6, 0.1 mM EDTA, 40 mM KCl, 10% glycerol, 1 mM dithiothreitol, 1 mg/mL leupeptin, 1 mg/mL pepstatin, 0.1 mM phenylmethanesulphonylfluoride (PMSF), 1% aprotinin, 1 mM benzamidine).

### Dual luciferase assay

Approximately 24 h prior to transfection, cells were plated in 12-well plates at 1 × 10^5 ^cells/well. Cells were transfected in triplicate using a total of 250 ng DNA per well with 0.75 μL/well FuGENE (Roche, Laval, Canada) according to the manufacturer's protocol. The specific amounts of material used per well were: 25 ng of the CMV-luc internal control, 25 ng of each expression vector or empty vector control, 50 ng of shRNA plasmid and a *Renilla *luciferase reporter vector up to a total of 250 ng. Approximately 48 h post-transfection, the cells were washed with phosphate buffered saline (PBS), lysed in 150 μL passive lysis buffer (Promega), and 20 μL of the cell lysates were assayed using the Dual-Luciferase® Reporter Assay System according to the manufacturer's instructions (Promega) with a EG&G Berthold microplate luminometer.

### Electrophoretic mobility shift assay (EMSA)

Nuclear extracts were prepared as previously described [17] with the exception that nuclear proteins were not concentrated by (NH_3_)_2_SO_4 _precipitation, but were dialyzed against 10 mM HEPES pH 7.6, 40 mM KCl, 0.1 mM EDTA, and 10% glycerol. Nuclear extracts or recombinant protein (2-4 μg unless otherwise indicated) were combined with ^32 ^P-labelled oligonucleotides (1 ng) in binding buffer (25 mM HEPES pH 7.6, 5 mM MgCl_2_, 34 mM KCl, 50 μg/mL poly dI:dC (Sigma), 0.5 mg/mL bovine serum albumin (BSA)). Binding reactions (20 μL final volume) were incubated on ice for 15 min prior to separation on a 6% acrylamide 0.25 × TBE non-denaturing gel. For competition assays, unlabelled oligonucleotide competitors were mixed with ^32 ^P-labelled oligonucleotides in binding buffer prior to the addition of nuclear extracts. For the supershift assay, 2 μg anti-NRF-1 (ab34682, Abcam, Cambridge, MA, USA) or PBS (negative control) was incubated with 4 μg of nuclear extracts for 30 min on ice prior to the addition of ^32 ^P-labelled oligonucleotide in binding buffer. Oligonucleotide sequences are given in Figures 6 and 9 (positive strand only). The sequence of RC4, an oligonucleotide containing the NRF-1 binding sequence from the rat cytochrome C promoter (nucleotide -173 to -147), has been previously reported [22].

### Chromatin immunoprecipitation (ChIP)

ChIP assays were performed with the ChIP-IT™ Express kit according to the manufacturer's instructions (Active Motif, Carlsbad, CA, USA). Each immunoprecipitation reaction contained chromatin from 1.5 × 10^6 ^cells, and 2 μg of antibody (or water as a negative control). The following antibodies were used: acetylated histone H3K9 (06-599, Upstate Biotechnology, Lake Placid, NY, USA), haemagglutinin (Y-11, Santa Cruz Biotechnology, Santa Cruz, CA, USA), RNA polymerase II (Covance, Emeryville, CA, USA), histone deacetylase I (ab7028, Abcam), NRF-1 (ab34682, Abcam), and Oct-4 (ab19857, Abcam). PCR primers amplified the BRCA1 promoter from position -341 to +116 ((+) 5'-GATTGGGACCTCTTCTTACG and (-) 5'-TACCCAGAGCAGAGGGTGAA)) and the *GABPβ *promoter from position -358 to -178 ((+) 5'-CTCCTACCCACCGCAGAAC and (-) 5'-CCATTTCTAGCGCTTCAGCC). A water blank (no template) and the initial chromatin were also subjected to PCR amplification as controls.

### siRNA Knockdown

For dual luciferase assays involving siRNA knockdown, MCF-7 cells were plated at 5 × 10^4 ^cells/well in 24-well plates approximately 24 h prior to transfection. Cells were transfected in triplicate with siRNA (100 ng/well), *GABPβ *promoter constructs (175 ng/well), and CMV-luc (25 ng/well) for normalization of transfection efficiency, using TransMessenger™ Transfection Reagent (Qiagen, Mississauga, Canada) according to the manufacturer's protocol. Approximately 48 h post-transfection, the cells were washed with PBS, lysed in 75 μL passive lysis buffer and 20 μL of the cell lysates were assayed using the Dual-Luciferase® Reporter Assay System according to the manufacturer's recommendations. For Western blots, MCF-7 cells were plated at 2.5 × 10^5 ^cells/well in 6-well plates approximately 24 h prior to transfection. Cells were transfected with siRNA (1 μg per well) using Santa Cruz Transfection Reagent (Santa Cruz Biotechnology) according to the manufacturer's protocol. Approximately 72 h post-transfection, cells were washed twice with PBS and lysed in 200 μL loading buffer (2.5% SDS, 25 mM Tris-HCl pH 6.8, 10% glycerol, 1% apropotin, 1 mM dithiothreitol, 1 μg/mL leupeptin, 1 μg/mL pepstatin, 0.1 mM PMSF, 1 mM NaF, 1 mM sodium orthovanadate, 20 mM β-glycerophosphate). siRNA used: siGAPDH (siGENOME® GAPD Control siRNA, Thermo Scientific Dharmacon, Lafayette, CO, USA) and siNRF-1 (5'-CGUUAGAUGAAUAUACUACtt, Ambion, Austin, TX, USA) [27].

### Semi-quantitative RT-PCR

RNA was isolated using the Genelute Mammalian Total RNA Miniprep Kit (Sigma). cDNA was generated by reverse-transcribing 2.5 μg of RNA for 5 min at 70°C and then 1 h at 42°C in a reaction mix containing 1 × MMluV reaction buffer (Invitrogen), 1 μg pol(N)_6 _primer (Pharmacia), 0.5 mM dNTPs, 1 μL RNAse OUT (Invitrogen), and 1 μL MMluV-RTase enzyme (Invitrogen) made up to 50 μL with diethylpyrocarbonate (DEPC)-treated water. Primer pairs specific to each of the GABP subunits and GAPDH were then used to amplify 2 μL of each RT product. In addition to the RT product and 500 ng of each primer, the reactions contained 1 × Thermopol buffer (NEB), 0.25 mM dNTPs, 1 μL Vent (NEB) and DEPC-treated water up to a final volume of 50 μL. The PCR protocol consisted of 4 min at 98°C, 29-33 cycles of (30 sec at 98°C, 1 min at 55°C, 1 min at 72°C) followed by 4 min at 72°C. Loading buffer was added to each sample to a final concentration of 2.5% Ficoll, 0.025% bromophenol blue and 0.1 mM EDTA, and 10 μL of each sample was resolved on a 1.5% agarose gel. Primers are specified in Additional File 2.

### Quantitative RT-PCR

RNA and RT products were prepared as described above. Quantitative RT-PCR reactions for BRCA1 (with TBP as an internal control) were performed using the SuperScript® III Platinum® One-Step Quantitative RT-PCR system (Invitrogen) with 500 ng RNA per reaction and LUX™ primers specified in Additional File 2 according to the manufacturer's instructions. The PCR protocol consisted of 1 cycle of (900 sec at 55°C and 120 sec at 95°C), followed by 40 cycles of (30 sec at 95°C, 30 sec at 55°C, 30 sec at 72°C). BRCA1 expression for each cell line was calculated relative to the results for the MCF-7 cell line using the Pfaffl method [28].

Quantitative RT-PCR reactions for GABPβ were performed using the QuantiTect SyBr Green PCR kit (Qiagen) with 2.5 μL of RT product as per the manufacturer's instructions. Primer pairs and annealing temperatures (Tm) are specified in Additional File 2. The PCR protocol consisted of 1 cycle of 900 sec at 95°C followed by 45 cycles of (15 sec at 95°C, 30 sec at Tm°C, 30 sec at 72°C). GABPβ expression for each cell line was calculated relative to the results for the 184hTERT cell line using the delta-delta Ct method presented by PE Applied Biosystems (Perkin Elmer, Forster City, CA, USA).

### Preparation of whole cell lysates

For GABP subunit complementation assays, cells were plated as described for dual luciferase assays. Transfections were performed using 3 μL FuGENE transfection reagent and 1 μg of each expression plasmid per well (total of 2 μg DNA per well), as per the manufacturer's instructions. Forty-eight hours post-transfection, the cells were scraped using a rubber policeman and lysed using 50 μL/well modified RIPA buffer (50 mM Tris-HCL pH 7.4, 1% Igepal C630, 0.25% Na-deoxycholate, 150 mM NaCl, 1 mM EDTA, 1 mM PMSF, 1 μg/mL each of aprotinin, leupeptin and pepstatin, 1 mM sodium orthovanadate, 1 mM NaF) for 15 min at 4°C. An equal amount of 2 × SDS-PAGE loading buffer was added to each lysate. To determine the endogenous BRCA1, GABPα and GABPβ protein levels, cells were grow to 60% confluence, scraped using a rubber policeman and lysed using modified RIPA buffer for 15 min at 4°C. An equal amount of 2 × SDS-PAGE loading buffer was added to each lysate.

### Western blot

Whole cell lysates were resolved on a SDS-polyacrylamide gel, transferred to a nitrocellulose or PVDF membrane, and probed with the appropriate antibody. Primary antibodies included: anti-BRCA1 (0P92, 1:500, Calbiochem, San Diego, CA, USA), anti-GABPα (H-180, 1:500, Santa Cruz Biotechnology), anti-GABPβ (H-265, 10 ×, 1:5000, Santa Cruz Biotechnology), anti-FLAG (M2, 1:1000, Sigma), anti-NRF-1 (M01, 1:500, Abnova, Taipei, Taiwan), anti-γ-tubulin (GTU-88, 1:5000, Sigma), and anti-TBP (1TBP18, 1:2000, Abcam). Secondary antibody detection was performed by chemiluminescence (Thermo Scientific/Fisher, Nepean, Canada).

### Immunofluorescence

For immunofluorescence analysis of proteins, cells were plated on coverslips 24 h prior to transfection, in 12-well plates at a density of 1 × 10^5 ^cells/mL. Transfections were performed using 0.75 μL of FuGENE transfection reagent and 125 ng of each expression plasmid per well (total of 250 ng DNA per well), as per the manufacturer's instructions. Cells were incubated at 37°C for 48 h at which time the media was aspirated and the wells washed with PBS. The cells were fixed at room temperature using 4% paraformaldehyde in PBS, aspirated, washed with PBS, and then permeablized at room temperature using 0.5% TritonX-100 in PBS. The cells were incubated in blocking buffer (3% BSA, 10% Normal Goat Serum, 0.1% Triton X-100, 0.1% Tween 20 in PBS) at room temperature for one hour, followed by primary antibody solution (1:200 dilution of antibody in PBS, 3% BSA) at room temperature for one hour in a humidified chamber, and then washed with PBS. All steps were performed in the dark from this point onward. The coverslips were incubated in secondary antibody solution (1:100 dilution of antibody in PBS, 3% BSA) for one hour in the humidified chamber, washed with PBS, and then the nuclei were stained for 10 minutes with Hoechst in PBS. The coverslips were given a final wash with PBS and then mounted onto slides using Permount Anti-fade mounting medium. Images were visualized on a Leica TCS SP2 Multi Photon confocal microscope. FITC was excited using a 488 nm laser and detected at 525 nm ± 20, and Hoescht was excited using a 2-photon laser at 780 nm and detected at 450 nm ± 20. The imaging software used was Image Pro Plus.

## Results

### Differential expression of BRCA1 in MCF-7 verses SK-BR-3 cell lines

**Figure 1:**
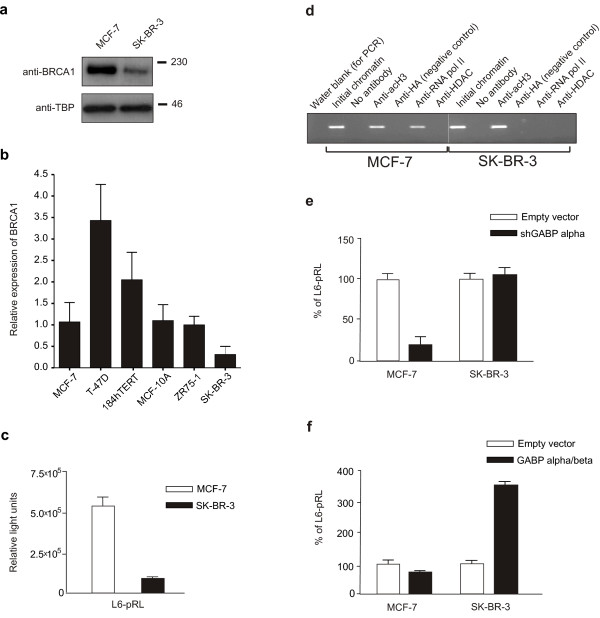
**BRCA1 expression and GABP alpha/beta activity is reduced in SK-BR-3 cells**. (**a**) Western blot analysis of BRCA1 protein levels in MCF-7 and SK-BR-3 cells. Equal quantities of proteins were loaded and levels of TATA Binding Protein (TBP) are shown as an internal control. (**b**) Quantitative RT-PCR for BRCA1 was carried out for a variety of breast cell lines using TBP as an internal control. Levels are shown relative to MCF-7 cells with the mean and standard deviation of three replicates shown. (**c**) The relative activities of the BRCA1 proximal promoter construct (L6-pRL) in MCF-7 and SK-BR-3 cells were compared using normalization with an internal control plasmid. For all transfection experiments reported here, the mean and standard deviation of 3 replicates are indicated. Independent experiments were performed a minimum of three times. (**d**) A ChIP assay was performed using MCF-7 and SK-BR-3 chromatin and antibodies against acetylated histone H3K9 (acH3), haemagglutinin (HA, negative control), RNA polymerase II (RNA pol II) and histone deacetylase I (HDAC). PCR products obtained using primers specific to the *BRCA1 *promoter (refer to Methods) are shown. (**e**) The BRCA1 L6-pRL construct was co-transfected with a small hairpin RNA expression construct directed against GABP alpha (shGABP alpha), or the empty H1-2 vector (Empty vector). Results are expressed in relation to the vector transfected cells, for each cell line. (**f**) Expression vectors for both GABP alpha and beta (GABP alpha/beta) were cotransfected with the L6-pRL promoter construct in both cell lines. Results are expressed in relation to the empty vector controls for each cell line.

Cell lines and tumours overexpressing ErbB2 have been reported to have particularly low BRCA1 levels [29,30]. This phenomenon was confirmed by comparison of the breast cancer cell lines MCF-7, which has low ErbB2 levels, and SK-BR-3, which highly overexpresses the receptor. Western blot analysis of whole cell lysates confirmed the low level of BRCA1 protein in SK-BR-3 cells (Figure 1a) and quantitative RT-PCR analysis of BRCA1 mRNA levels in multiple breast lines suggests this decrease may be due to limited transcription in SK-BR-3 cells (Figure 1b).

To investigate the molecular basis of low BRCA1 expression in the SK-BR-3 line, the activity of the *BRCA1 *proximal promoter (*i.e*. L6-pRL) [24] was examined in the two lines. When the activities of the constructs were normalized using a dual luciferase assay, expression was approximately 6-fold lower in the SK-BR-3 line compared to MCF-7 cells (Figure 1c). Consistent with this observation, chromatin immunoprecipitation (ChIP) revealed that the *BRCA1 *promoter is occupied by RNA polymerase II (RNA pol II) in MCF-7 but not SK-BR-3 cells, suggesting that the promoter is transcribed at a low level in the SK-BR-3 cell line (Figure 1d). The lack of histone deacetylase I (HDAC) binding suggests this protein is not involved in *BRCA1 *downregulation (Figure 1d).

Having previously demonstrated that the multi-subunit *ets *transcription factor GABP is a critical regulator of the *BRCA1 *proximal promoter [17], the effects of modulation of GABP levels on *BRCA1 *promoter activity were evaluated. Co-transfection of a shRNA construct directed against the alpha subunit of GABP resulted in a dramatic decrease in *BRCA1 *promoter activity in MCF-7s, but had no effect on its activity in the SK-BR-3 line (Figure 1e). Overexpression of the GABP alpha and beta subunits however, had no effect on promoter activity in MCF-7s (Figure 1f), presumably due to the presence of saturating endogenous levels of these proteins. In contrast, cotransfection of these expression vectors resulted in a dramatic increase in the transcriptional activity of the L6-pRL promoter in SK-BR-3 cells (Figure 1f). These results suggest that endogenous GABP is either absent or non-functional in the SK-BR-3 cell line and that this loss is responsible for the low level of BRCA1 expression in this line.

### Endogenous GABPβ activity and levels are lower in SK-BR-3 cells

**Figure 2:**
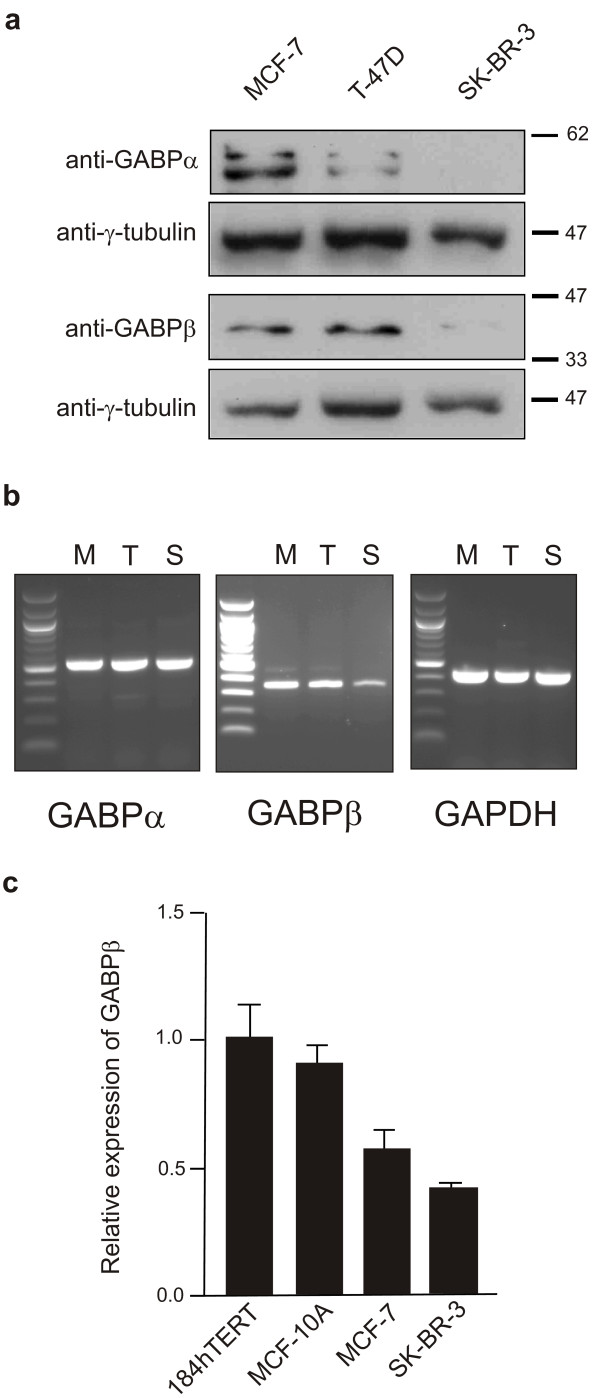
**GABPα and β subunit protein and mRNA levels are decreased in the SK-BR-3 cell line**. (**a**) Western blot analysis of whole cell lysates from MCF-7, T-47D and SK-BR-3 cells was carried out using antibodies to GABPα, GABPβ and the blots were then reprobed with anti-γ-tubulin as an internal control. Apparent molecular weight markers (kDa) are indicated to the right of the panels. (**b**) The relative transcript levels of the GABP subunits in MCF-7 (M), T-47D (T) and SK-BR-3 (S) cells were examined by semi-quantitative RT-PCR. Specific products were amplified from equal amounts of RT product from the cell lines indicated using primer sets for GABPα, GABPβ and GAPDH as an internal control. Products were separated on a 1.5% agarose gel with 100 bp ladder in leftmost lane. (**c**) Quantitative RT-PCR analysis of GABP beta-41 subunit mRNA was carried out on the indicated cell lines. Levels are expressed in relation to the 184hTERT cell line.

To determine if GABP protein levels were altered in SK-BR-3 cells, cell lysates from three breast cell lines, MCF-7, T-47D and SK-BR-3, were evaluated by Western blot. Equal quantities of total protein from both nuclear (data not shown) and whole cell extracts (Figure 2a) were analyzed. Levels of both GABPα and GABPβ were dramatically reduced in SK-BR-3 cells compared to MCF-7 cells and T-47D cells, though some reduction in the levels of these proteins in T-47D cells was noted (Figure 2a). Semi-quantitative RT-PCR was then carried out on all three cell lines using PCR primers directed against the alpha and beta subunits, as well as GAPDH as an internal control. The alpha subunit mRNA appears to be expressed at similar levels in all three cell lines while the beta form is significantly reduced in SK-BR-3 cells (Figure 2b). The reduced levels of GABPβ mRNA in SK-BR-3 cells were confirmed by quantitative RT-PCR (Figure 2c). These results suggest that the low levels of GABPβ protein in the SK-BR-3 cell line are the result of a lack of expression of the beta gene. While the GABPα mRNA levels are similar, the low levels of GABPα protein may be attributable to the lack of its binding partner.

### Expression of exogenous GABPβ restores BRCA1 proximal promoter activity, and GABPα levels and localization in SK-BR-3 cells

**Figure 3:**
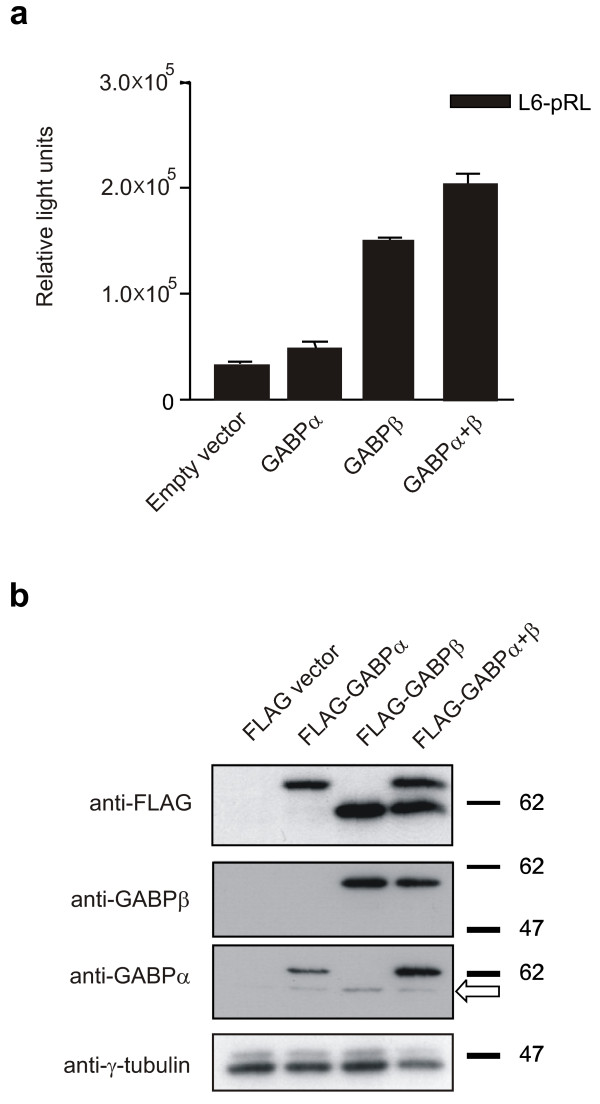
**Exogenous GABPβ in SK-BR-3 cells restores BRCA1 proximal promoter activity and stabilizes endogenous GABPα**. (**a**) Expression vectors for the individual GABP subunits, or both together, were cotransfected with the BRCA1 L6-pRL promoter construct in SK-BR-3 cells. (**b**) SK-BR-3 cells were co-transfected with the indicated FLAG-tagged GABP expression vectors. Whole cell lysates from these cells were analyzed by Western blots probed with antibodies against GABPα, GABPβ or the FLAG moiety and then reprobed with anti-γ-tubulin to control for sample loading. The arrow indicates the band corresponding to endogenous GABPα protein. Apparent molecular weight markers (kDa) are presented to the right of the panels.

These results suggest that GABPβ expression is compromised and was confirmed when cotransfection of the beta subunit alone, but not the alpha subunit, was able to transactivate the *BRCA1 *promoter in SK-BR-3 cells (Figure 3a). To confirm these results, whole cell lysates from SK-BR-3 cells transfected with FLAG-tagged expression vectors for GABPα and GABPβ were evaluated by Western blot to determine the relative levels of GABP subunit expression (Figure 3b). The FLAG-GABPα levels were also increased by the presence of FLAG-GABPβ confirming that alpha protein is stabilized by the presence of its partner. Endogenous GABPα levels were almost undetectable in the lysates from cells transfected with the empty FLAG vector (Figure 3b, FLAG vector, anti-GABPα), the arrow indicates endogenous GABPα. Cells transfected with the FLAG-GABPβ expression vector however, produced detectable amounts of endogenous GABPα protein, confirming that exogenous GABPβ protein is able to stabilize endogenous GABPα expression (Figure 3b, FLAG-GABPβ, anti-GABPα), while endogenous GABPβ remains undetectable in all cases. No effect on endogenous BRCA1 levels was detected following exogenous GABPα and/or GABPβ expression (data not shown) likely due to the initial regulation defect leading to permanent downregulation of BRCA1 in this cell line consistent with the low level of RNA pol II detected by ChIP (Figure 1d).

**Figure 4:**
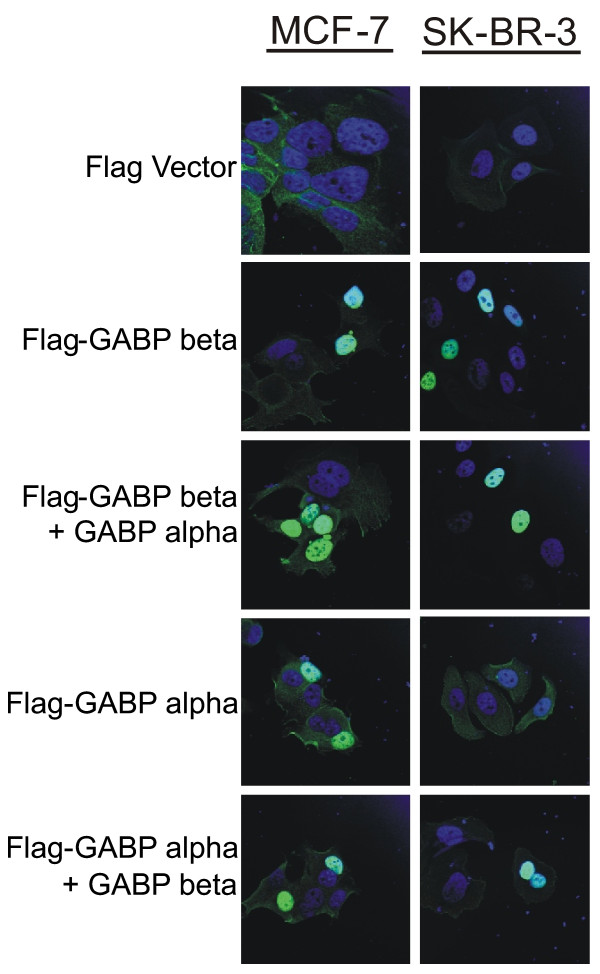
**GABPα nuclear localization is rescued by GABPβ in SK-BR-3 cells**. MCF-7 and SK-BR-3 cells were transfected with the indicated expression vectors. Cells were stained with anti-FLAG FITC-labeled antibodies (green) and Hoechst dye (blue). Confocal imaging of the overlay of the two stains is shown.

Translocation of GABPα into the nucleus is dependent on a nuclear localization signal present in GABPβ [21]. The effect of beta levels on alpha translocation was determined by transfection of the FLAG-tagged GABPα construct into MCF-7 and SK-BR-3 cells and visualization of the proteins using confocal microscopy with antibodies against the FLAG moiety. In MCF-7 cells, the alpha protein is present in the nucleus and addition of an expression vector for GABPβ does not alter its location (Figure 4, FLAG-GABP alpha, FLAG-GABP alpha + GABP beta). In contrast, the alpha subunit is present in the cytoplasm in SK-BR-3 cells (Figure 4, FLAG-GABP alpha), presumably due to the lack of GABPβ. Addition of a GABPβ expression vector causes the alpha protein to translocate into the nucleus (Figure 4, FLAG-GABP alpha + GABP beta). This confirms the absence of beta in SK-BR-3 cells and indicates that nuclear localization of the alpha protein can be rescued by the addition of exogenous GABPβ.

Thus, the decreased expression of BRCA1 in SK-BR-3 cells appears to be the result of a loss of GABPβ expression, destabilizing the α/β heterodimer and in turn leading to decreased BRCA1 expression due to the absence of GABP-mediated activation of the BRCA1 promoter.

### A critical activating factor(s) binds to the GABPβ promoter between -268 and -251

**Figure 5:**
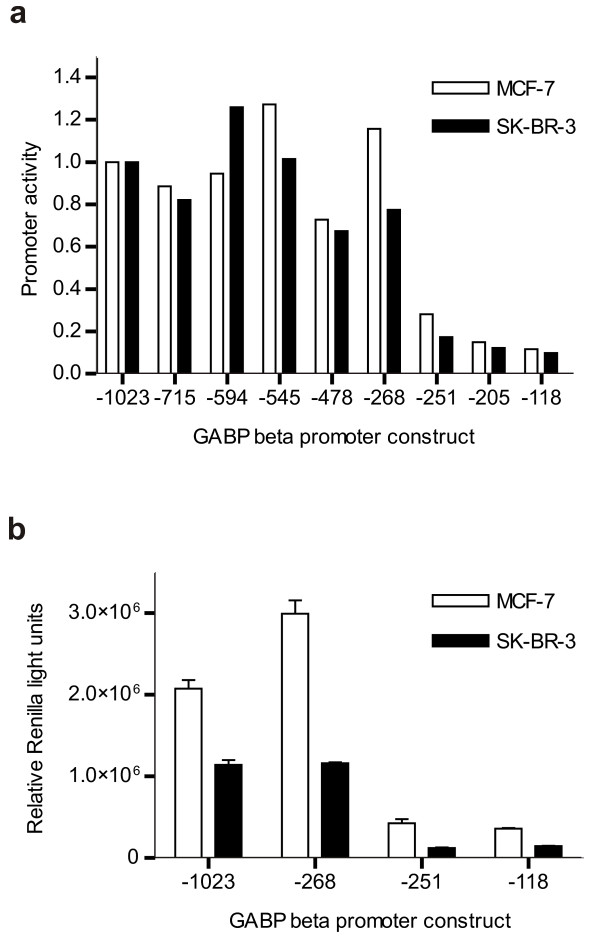
***GABPβ *promoter activity in MCF-7 and SK-BR-3 cell lines**. A series of 5' deletion mutants of the *GABPβ *proximal promoter were prepared in the pRL-null reporter plasmid. Promoter constructs are named according to the 5' nucleotide position relative to the transcription start site with all constructs extending to nucleotide +194. The transcriptional activity of the *GABPβ *promoter constructs was assessed via dual luciferase assay using the pCMV-luc plasmid as an internal control. (**a**) Promoter activity is expressed relative to the activity of the longest construct,-1023. (**b**) SK-BR-3 raw *Renilla *light units were multiplied by a correction factor to compensate for differences in transfection efficiency and permit a comparison of the absolute promoter activity between the two cell lines (Relative *Renilla *light units). The correction factor was based upon the luciferase light units (LLU) of the internal control, pCMV-luc, and was calculated by dividing the mean MCF-7 LLU by the mean SK-BR-3 LLU for the longest *GABPβ *promoter construct,-1023.

In order to characterize the basis for the downregulation of *GABPβ *in SK-BR-3 cells, the proximal promoter from -1023 to +194 was cloned and a series of deletion constructs were prepared using a *Renilla *luciferase reporter plasmid. These constructs identified a decrease in activity when the sequence between positions -268 and -251 was deleted in both MCF-7 and SK-BR-3 cells (Figure 5a). This suggested that a critical activating factor(s) binds to this site. Adjusting the *Renilla *light units to compensate for differences in transfection efficiency to permit a comparison of the absolute promoter activity between the two cell lines, revealed a reduction (approximately 2 to 3.5-fold) in *GABPβ *promoter activity in SK-BR-3 cells compared to MCF-7 cells (Figure 5b), although both cell lines showed similar deletion profiles. This indicates that the promoter is less active in SK-BR-3 cells, but the principle transcription factor(s) required for promoter activity is functional in both cell lines.

**Figure 6:**
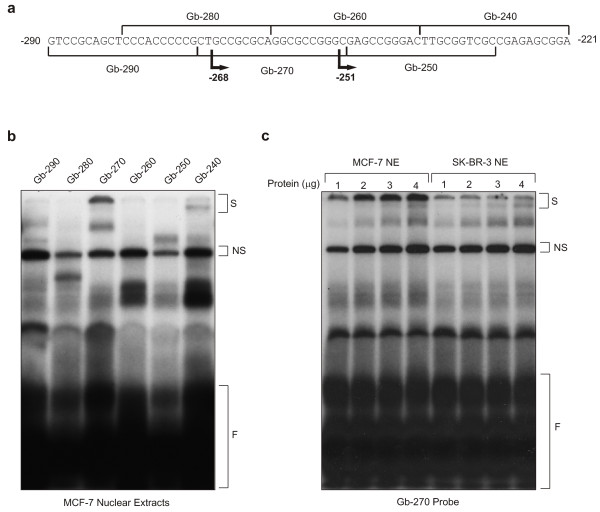
**Binding complexes that form on the *GABPβ *promoter**. (**a**) A series of overlapping 20-mer oligonucleotides (sequences specified by square brackets) spanning the *GABPβ *promoter from nucleotide -290 to -221 were prepared to examine the binding complexes that form on the promoter. The deletion constructs that border the sequence critical for promoter activity (Figure 5) are indicated (arrows, bold). (**b**) The 20-mer oligonucleotides were used as probes in an EMSA with nuclear extracts from MCF-7 cells. Binding complexes are indicated (Shift = S), as are non-specific binding complexes (NS) and free probe (F). (**c**) Gb-270 was used as a probe in an EMSA with increasing amounts of nuclear extracts (NE) from MCF-7 and SK-BR-3 cells. Binding complexes are as indicated above.

To confirm binding of a critical activating factor(s) to this region of the promoter, six overlapping 20-mer oligonucleotides representing the *GABPβ *promoter from position -290 to -221 were synthesized (Figure 6a) and assessed in an electrophoretic mobility shift assay (EMSA) with MCF-7 nuclear extracts (Figure 6b). Different binding complexes for each oligonucleotide were observed including a non-specific binding complex (NS) on each oligonucleotide, a weak doublet on Gb-240 (S), and a large binding complex on Gb-270 (S), which encompasses the critical sequence identified by deletion analysis ( -268 to -251). Interestingly, the complex that forms on Gb-270 differs between MCF-7 and SK-BR-3 cells (Figure 6c). While a robust single band representing a large binding complex was formed with MCF-7 nuclear extracts, a weaker doublet was formed with the same amount of SK-BR-3 nuclear extracts by weight (with normalization verified via the non-specific binding complex, NS). It is possible that this difference in the levels of binding proteins is responsible for the lower *GABPβ *promoter activity observed in SK-BR-3 cells (Figure 5b).

### NRF-1 binds to the GABPβ promoter between -268 to -251

**Figure 7:**
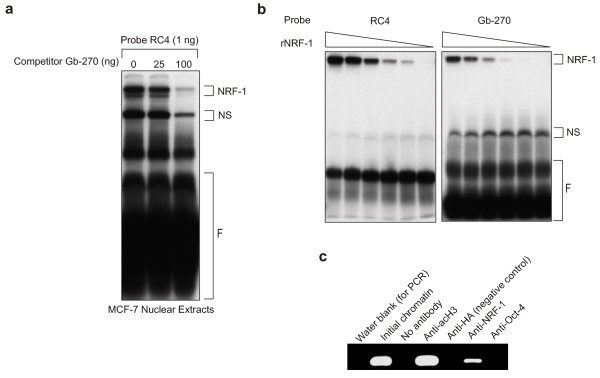
**NRF-1 binds to the *GABPβ *promoter**. (**a**) An oligonucleotide with a known NRF-1 binding site (RC4) [22] was used as a probe in an EMSA with MCF-7 nuclear extracts and Gb-270 as a cold competitor. NRF-1 binding (NRF-1), non-specific (NS) binding and free probe (F) are indicated. (**b**) Recombinant NRF-1 (rNRF-1) was prepared as a fusion with the maltose binding protein. Decreasing amounts of recombinant protein (5, 2.6, 1.3, 0.26, 0.13 and 0.03 μg) were used in an EMSA with RC4 and Gb-270 probes. Binding complexes as indicated above. (**c**) A ChIP assay was performed using MCF-7 chromatin and antibodies against acetylated histone H3K9 (acH3), haemagglutinin (HA, negative control), NRF-1 and Oct-4 (transcription factor, negative control). PCR products obtained using primers specific to the *GABPβ *promoter (refer to Methods) are shown.

Analysis of the *GABPβ *promoter sequence between -268 and -251 revealed its similarity to the consensus binding sequence of NRF-1 [22]. Given that GABP and NRF-1 are key regulators of mitochondrial respiration [31], this suggested a potential linkage in their regulation. Binding of NRF-1 to the *GABPβ *promoter was initially demonstrated in an EMSA in which Gb-270 was able to compete in a dose-dependent fashion for NRF-1 binding with RC4, an oligonucleotide containing the NRF-1 binding site from the rat cytochrome C promoter [22] (Figure 7a). This result was verified by an EMSA in which recombinant NRF-1, prepared and purified as a fusion with maltose binding protein, bound in a concentration-dependent manner to both Gb-270 and RC4 (Figure 7b). Finally, binding of NRF-1 to the *GABPβ *promoter *in vivo *was verified by ChIP (Figure 7c). MCF-7 chromatin was immunoprecipitated with a variety of antibodies and after PCR amplification of the proximal promoter, the antibody against NRF-1 gave a positive signal, while negative controls did not. Together, the EMSA and ChIP results confirm that NRF-1 binds to the *GABPβ *promoter between -268 and -251 *in vitro *and *in vivo*.

### Loss of NRF-1 decreases GABPβ and BRCA1 gene expression

**Figure 8:**
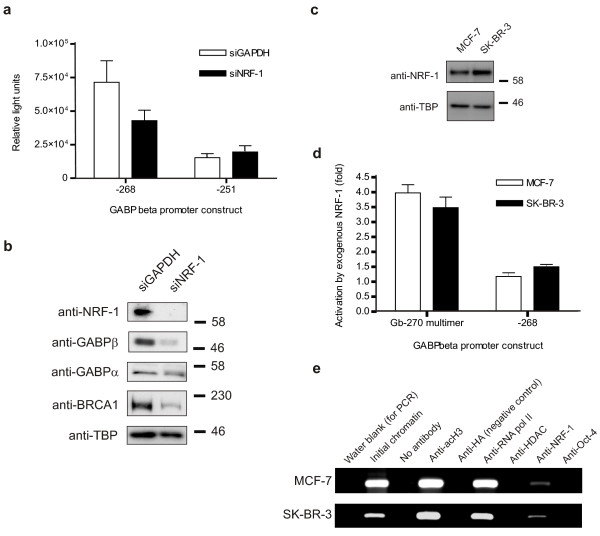
**NRF-1 loss attenuates *GABPβ *promoter activity and GABPβ/BRCA1 expression; NRF-1 is consistent between cell lines**. (**a**) The transcriptional activity of the *GABPβ *promoter constructs -268, which contains the NRF-1 binding site, and -251 which does not, was assessed in MCF-7 cells via dual luciferase assay in the presence of siRNA against GAPDH (siGAPDH, negative control) and NRF-1 (siNRF-1). Promoter activity is expressed as relative light units. (**b**) The protein levels of NRF-1, GABPβ, GABPα, BRCA1 and TBP (internal control) were assessed by Western blot in whole cell lysates prepared from MCF-7 cells treated with siGAPDH or siNRF-1. (**c**) NRF-1 levels were determined by Western blot in MCF-7 and SK-BR-3 whole cell lysates. TBP was used as an internal control. Apparent molecular weight markers (kDa) are indicated to the right of the panels. (**d**) The activity of two *GABPβ *promoter constructs, Gb-270 multimer (which contains a triple repeat of the Gb-270 sequence specified in Figure 6) and -268 (see part **a**), was examined via dual luciferase assay in MCF-7 and SK-BR-3 cells following exogenous NRF-1 expression. Promoter activation by NRF-1 is expressed as a fold relative to empty vector controls in each cell line. (**e**) A ChIP assay was performed using MCF-7 and SK-BR-3 chromatin and antibodies against acetylated histone H3K9 (acH3), haemagglutinin (HA, negative control), RNA polymerase II (RNA pol II), histone deacetylase I (HDAC), NRF-1 and Oct-4 (transcription factor, negative control). PCR products obtained using primers specific to the *GABPβ *promoter (refer to Methods) are shown.

To investigate the role of NRF-1 on the *GABPβ *promoter, MCF-7 cells were transfected with siRNA against NRF-1 or GAPDH (as a negative control), and one of two *GABPβ *promoter constructs, -268 which contains the NRF-1 binding site, and -251 which does not (Figure 8a). Knockdown of NRF-1 attenuated the promoter activity of -268, but not -251, indicating that loss of NRF-1 binding decreases *GABPβ *transcription and depends on this promoter region. The effects of NRF-1 knockdown were confirmed in a Western blot on whole cell lysates from MCF-7 cells transfected with siGAPDH or siNRF-1 (Figure 8b). NRF-1 was reduced to undetectable levels, while GABPβ was decreased to approximately 40% (normalized with the loading control, TBP). Interestingly, the levels of GABPα were unaffected by NRF-1 knockdown, although the change in band appearance (*i.e*. single band to a doublet) could indicate an alteration in post-translational modification (Figure 8b). BRCA1 protein levels were also decreased by the NRF-1 siRNA indicating that it lies downstream of both GABP and NRF-1, forming a transcriptionally regulated network.

### NRF-1 levels and activity are similar between MCF-7 and SK-BR-3 cells

Given that NRF-1 binds to and regulates the *GABPβ *promoter (Figure 7, 8a, b), it was important to determine if low *GABPβ *expression in SK-BR-3 cells could be the result of decreased NRF-1 levels. Western blot analysis demonstrated that the levels of NRF-1 between MCF-7 and SK-BR-3 cells are similar (Figure 8c), and thus, are unlikely to be responsible for low *GABPβ *expression in SK-BR-3 cells. It was also important to ascertain whether NRF-1 activity was compromised in SK-BR-3 cells. MCF-7 and SK-BR-3 cells transfected with an NRF-1 expression vector (p3×FLAG-NRF-1) and one of two *GABPβ *promoter constructs, Gb-270 multimer (which contains a triple repeat of the Gb-270 sequence specified in Figure 6) and -268 (as referenced above), were assessed in a dual luciferase assay. Activation of the promoter constructs by exogenous NRF-1 was similar in the MCF-7 and SK-BR-3 lines confirming that NRF-1 function was not defective in SK-BR-3 cells (Figure 8d). In addition, ChIP analysis revealed that the *GABPβ *promoter is active (as evidenced by the presence of acetylated histone H3K9 (acH3) and RNA pol II, and the lack of HDAC) and occupied by NRF-1 in both cell lines (Figure 8e). Thus, altered NRF-1 activity does not appear to account for the discrepancy in *GABPβ *expression between MCF-7 and SK-BR-3 cells.

### NRF-1 is one member of a protein complex that activates GABPβ transcription

**Figure 9:**
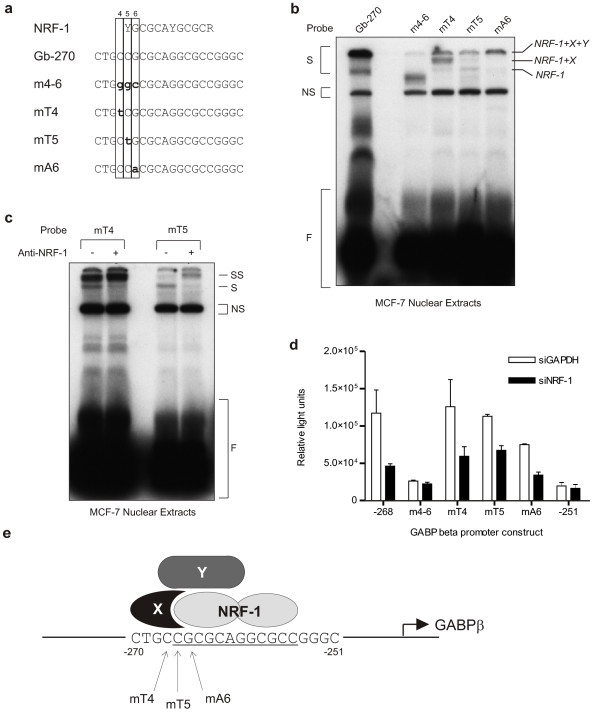
**NRF-1 is a component of a protein complex that binds to the *GABPβ *promoter**. (**a**) The NRF-1 consensus sequence [22], and the sequences of Gb-270 and mutant oligos are shown. Mutations are in lowercase and bold. (**b**) Gb-270 and the mutant oligos were used as probes in an EMSA with MCF-7 nuclear extracts. Binding complexes (S), non-specific binding complexes (NS) and free probe (F) are indicated. Bands corresponding to NRF-1, and predicted NRF-1 + X and NRF-1 + X +Y complexes (see part **e**) are also indicated. (**c**) Mutant oligos mT4 and mT5 were used as probes in an EMSA with MCF-7 nuclear extracts. An NRF-1 antibody was added to the binding reactions as indicated (+), or PBS was added as a negative control (-). Binding complexes (S) were supershifted (SS) in the presence of the NRF-1 antibody. (**d**) The transcriptional activities of the *GABPβ *promoter constructs -268 (contains the NRF-1 binding site) and -251 as well as promoter constructs with the mutations specified in part (**a**) were assessed in MCF-7 cells via dual luciferase assay in the presence of siRNA against GAPDH (siGAPDH, negative control) and NRF-1 (siNRF-1). Promoter activity expressed as relative light units. (**e**) Model of the NRF-1 complex that activates GABPβ gene expression. Banding patterns observed by EMSA suggest that NRF-1 binds to the *GABPβ *promoter in complex with at least two other proteins (refer to part **b**). We propose that NRF-1 binds as a homodimer to the *GABPβ *promoter from nucleotide -266 to -255 (underlined). Protein X binds NRF-1 and makes limited contact with the promoter upstream of the NRF-1 binding site stabilizing its interactions with NRF-1. A third protein (Y) binds to the NRF-1 + X complex. Mutations to the promoter (mT4, mT5 and mA6) disrupt formation of the complex (refer to part **b**), but oligonucleotides with mT4 and mT5 mutations retain NRF-1 binding capability (refer to part **c**).

Binding of NRF-1 to the *GABPβ *promoter was further characterized by evaluating a series of mutant oligonucleotides in an EMSA with MCF-7 nuclear extracts. The consensus binding sequence for NRF-1 [22] indicated that the NRF-1 binding site in Gb-270 began at nucleotide 5 (Figure 9a). Therefore, mutant versions of Gb-270 were prepared with conservative nucleotide replacements (*i.e*. C to G and G to C) at positions 4-6 (m4-6), and non-conservative single nucleotide replacements (*i.e*. C to T and G to A) at positions 4, 5 and 6 (mT4, mT5, mA6) (Figure 9a). Mutation of nucleotides 4-6 disrupted the large protein complex that normally forms on Gb-270 (Figure 9b, m4-6), whereas single nucleotide replacements at positions 4, 5 and 6 diminished the formation of the large protein complex and, in the case of positions 4 and 5, yielded faster migrating complexes as well (Figure 9b, mT4, mT5, mA6). A supershift EMSA with oligonucleotides mT4 and mT5 were used to probe MCF-7 nuclear extracts in the absence and presence of an antibody against NRF-1 (Figure 9c). Addition of the anti-NRF-1 antibody shifted the faster migrating complex (S) to a slower migrating complex (SS) for both oligonucleotides confirming that the faster migrating complex contained NRF-1. The fact that NRF-1 binding produces a faster migrating complex than what is normally observed on Gb-270 strongly suggests that the complex that forms on Gb-270 is actually a larger protein complex containing NRF-1. This is further supported by the faster migration of recombinant NRF-1 bound to Gb-270 (Figure 7b). The banding pattern observed (Figure 9b) suggests that NRF-1 binds to the *GABPβ *promoter in complex with at least two other proteins (Figure 9e).

To verify the role of NRF-1 on the *GABPβ *promoter, the mutants described were incorporated into the -268 promoter construct and these plasmids tested in co-transfection experiments in MCF-7 cells. As previously observed (Figure 8a), knockdown of NRF-1 attenuated the promoter activity of -268, which contains the NRF-1 binding site, but had no effect on the minimal activity of -251, which does not contain the NRF-1 site (Figure 9d). Mutation of nucleotides 4-6 decreased the *GABPβ *promoter activity to levels similar to -251 (m4-6) consistent with the disruption of the large protein complex previously observed by EMSA (Figure 9b, m4-6). Furthermore, knockdown of NRF-1 using a siRNA had no effect on the activity of this construct indicating that NRF-1 no longer binds and activates the *GABPβ *promoter. Mutation of nucleotides 4 and 5 did not decrease *GABPβ *promoter activity (Figure 9d, mT4, mT5) despite the diminished full protein complex formation observed by EMSA (Figure 9b). The promoter activities of mT4 and mT5 were attenuated by NRF-1 knockdown (Figure 9d) consistent with their ability to bind NRF-1 (Figure 9c). Interestingly, mA6, which showed diminished full complex formation but no uncomplexed NRF-1 binding (Figure 9b), exhibited reduced promoter activity that was also attenuated by NRF-1 knockdown (Figure 9d). This suggests that mutation of nucleotide 6 allowed binding of the full NRF-1-containing complex but with a reduced affinity. In summary, nucleotides 4-6 are required for assembly of the multi-protein complex, while binding of NRF-1 is required and sufficient for full promoter activity *in vitro*. The ability of the multi-protein complex to form in the presence of a point mutant in the NRF-1 site, though with lower affinity (as exemplified by mA6), as well as the decreased binding to Gb-270 observed in SK-BR-3 cells (Figure 6c) suggests the NRF-1 may bind co-operatively with other proteins (Figure 9e).

## Discussion

Like many ErbB2-overexpressing tumours [29,30], the SK-BR-3 cell line has low levels of BRCA1 protein and mRNA. In searching for the basic cause of this defect, we determined that the beta subunit of GABP, a key transcriptional regulator of the BRCA1 promoter [17], is itself downregulated. Decreased GABPβ activity is in turn linked to defects in an NRF-1/coactivator complex present on the *GABPβ *promoter. Knockdown of NRF-1 in MCF-7 cells confirms that this represents a NRF-1 > GABP > BRCA1 regulatory pathway. Given the inverse correlation between BRCA1 and ErbB2 levels in tumours, it was expected that some component of the NRF-1 > GABP > BRCA1 pathway would be sensitive to ErbB2-overexpression. However, cotransfection and siRNA knockdown experiments with both ErbB2 and all of the other Erb family members failed to affect the expression of any of these genes in both MCF-7 and SK-BR-3 lines (data not shown). This suggests that inactivation of this pathway is not a direct consequence of ErbB2 overexpression.

Both NRF-1 and GABP are known to control the expression of a wide variety of nuclear encoded mitochondrial proteins (reviewed in [31]). These include proteins involved in electron transport, such as cytochrome C and the cytochrome oxidases, as well as proteins involved in mitochondrial replication and maintenance. The expression of NRF-1 and GABP appear to be coordinated during mitochondrial biogenesis [31], but the basis for this has not previously been investigated. Our discovery of the presence of a functional and key NRF-1 regulatory element within the *GABPβ *promoter provides a molecular mechanism to explain this linkage. Because GABP is an obligate heterodimer [32], the GABPα protein levels must be coordinated with GABPβ levels. The *GABPα *promoter has previously been shown to be autoregulated [33], and GABPα levels in heterozygous knockout mice are the same as the wildtype indicating that protein levels are under tight control [34]. We have demonstrated that in the absence of GABPβ, GABPα protein is made but is unstable due to the lack of its partner, possibly as a result of changes in post-translational modification as seen with the siNRF-1 experiment (Figure 8b). This suggests that levels of GABPβ may be limiting and regulated, with GABPα being both stabilized and transcriptionally upregulated as GABPβ levels increase. This arrangement suggests that a positive feedback switch may exist, with GABP either lying downstream of NRF-1, or with NRF-1 also being regulated by GABP. Consistent with this, ChIP on CHIP analysis has located a GABP site in the proximal promoter of the NRF-1 gene [35]. The observation that BRCA1 may also be involved in negative autoregulation of its own promoter means that it could also participate in this feedback loop [36]. The formation of the NRF-1 complex on the *GABPβ *promoter, which differs between cell lines (Figure 6), was also shown to be dependent on the interaction of a coactivator complex with DNA sequences adjacent to the NRF-1 site (Figure 9). Because the induction of mitochondrial activity is controlled by the co-activator PGC1α which acts in conjunction with NRF-1 and GABP in muscle and fat tissue (reviewed in [37]), PGC1α was a candidate for the complex observed on the *GABPβ *promoter. We have been unable to observe any role for this coactivator in the induction of GABP function (data not shown). This has included cotransfection, siRNA and western blot analysis which indicate that neither PGC1α, PGC1β nor PRC are active or present in a variety of breast cell lines (data not shown). Indeed we have identified a different class of coactivators which are associated with NRF-1, and which could control mitochondrial biogenesis in these cells.

The identification of BRCA1 as a stem cell regulator in mammary cells [3] has expanded its already extensive list of possible functions. Based on this role, the frequent downregulation of BRCA1 expression seen in sporadic breast cancers could reflect the disruption of a stem cell differentiation program. Our findings suggest that BRCA1 is at the end of a transcriptional regulatory network consisting of NRF-1 and GABP. GABPα has been shown to be necessary for early embryonic growth with the homozygous knockout leading to death of the pre-implantation embryo [34]. The complete GABPβ knockout also exhibits early embryonic lethality [38], so that any defect in the GABP complex inhibits embryonic development. Interestingly, the NRF-1 knockout exhibits a similar phenotype [39], as would be expected if it was part of a common pathway with GABP. This pre-implantation phase of development is associated with a burst of mitochondrial synthesis [40]. BRCA1 knockouts are also embryonic lethal, but at a slightly later stage [41] suggesting that BRCA1 may lie downstream of both NRF-1 and GABP during embryogenesis. GABP has previously been implicated in the regulation of stem cells as a downstream target of STAT3, and ectopic expression of GABPα in embryonic stem cells activates Oct3/4 transcription by downregulating repressors of Oct3/4 expression [42]. In addition, bioinformatic analysis of stemness genes had previously implicated GABP in the regulation of stem cell proliferation [43]. This strongly suggests that GABP is linked to the regulation of stemness, in both the embryo and adult. At the same time, the linkage of NRF-1 and GABP to mitochondrial metabolism implies that BRCA1 expression, and thus the regulation of stemness, may also be linked to the activation of oxidative phosphorylation in these cells. The Warburg effect suggests that most cancers have a defect in oxidative phosphorylation which results in tumours primarily consuming glucose and producing lactic acid as the endpoint of metabolism [44]. Stem cells, and cancer stem cells, have also been suggested to be dependent on glycolysis [45]. If differentiation in the breast is linked by BRCA1 to the induction of mitochondrial metabolism, then blockade of this pathway will lead to both the persistence of stem-like properties and the lack of oxidative phosphorylation. The Warburg effect can then be viewed as the persistence of a metabolic program present in stem cells into the tumour state. Explanations of the Warburg effect have focused on alterations in proteins involved in mitochondrial function and uncoupling [46]. These changes must be underlaid by alterations in transcriptional regulation, presumably in networks such as NRF-1 and GABP involved in upregulating oxidative phosphorylation. Many of the pathways previously shown to be affected may represent compensatory activation of alternative metabolic pathways used by the cell to overcome defective induction of oxidative phosphorylation.

## Conclusions

In summary, recent evidence suggests that loss of BRCA1 function impairs normal breast differentiation thereby facilitating tumour initiation. Investigation of low BRCA1 expression in the human breast cancer cell line SK-BR-3 revealed a transcriptional network consisting of NRF-1 > GABPβ > BRCA1. Given the common role of NRF-1 and GABP in regulating mitochondrial function, the NRF-1 > GABPβ > BRCA1 pathway suggests a link between tumour initiation via disruption of stem cell maturation and the abnormal mitochondrial metabolism (Warburg effect) that has long been observed in tumours.

## List of abbreviations

BRCA1, breast cancer 1 early onset; BSA, bovine serum albumin; ChIP, chromatin immunoprecipitation; EMSA, electrophoretic mobility shift assay; ERα, estrogen receptor α; ErbB2, v-erb-b2 erythroblastic leukemia viral oncogene homolog 2; GABP, GA-binding protein; GAPDH, glyceraldehyde 3-phosphate dehydrogenase; HDAC, histone deacetylase I; NRF-1, nuclear respiratory factor-1; PBS, phosphate buffered saline; PGC1α, peroxisome proliferator-activated receptor gamma coactivator 1α; PGC1β, peroxisome proliferator-activated receptor gamma coactivator 1β; PMSF, phenylmethanesulphonylfluoride; PR, progesterone receptor; PRC, PGC-1-related coactivator; RNA pol II, RNA polymerase II; shRNA, small hairpin RNA; siRNA, small interfering RNA; STAT3, signal transducer and activator of transcription 3.

## Competing interests

The authors declare that they have no competing interests.

## Authors' contributions

CT and GM participated in the study design and carried out the experiments. CT drafted the manuscript. CRM conceived of the study and participated in its design and helped to draft the manuscript. All authors read and approved the final manuscript.

## Supplementary Material

Additional file 1 Excel Primers, templates and restriction enzymes used in the preparation of DNA constructs Details for the Methods. Click here for file

Additional file 2 Excel Primers used for RT PCR Details for the Methods. Click here for file
